# Molecular Characteristics and Survival Rates in the Patients with Gastrointestinal Stromal Tumors: A Retrospective Single-Center Study

**DOI:** 10.3390/cancers18203298

**Published:** 2026-10-13

**Authors:** Natalia N. Mazurenko, Irina V. Tsyganova, Vladimir V. Yugay, Olga A. Anurova, Ilya S. Beliakov, Anna A. Lushnikova, Maxim P. Nikulin, Nikolay A. Kozlov, Petr P. Arhiri, Daria A. Filonenko, Ivan S. Stilidi

**Affiliations:** 1 Research Institute of Experimental Oncology and Carcinogenesis, N.N. Blokhin National Medical Research Center of Oncology, Kashirskoye Sh. 24, 115478 Moscow, Russia; ivtsyganova@mail.ru (I.V.T.);; 2 Research Institute of Clinical Oncology, N.N. Blokhin National Medical Research Center of Oncology, Kashirskoye Sh. 24, 115478 Moscow, Russia; yugay_vladimir@mail.ru (V.V.Y.);; 3 A.S. Loginov Moscow Clinical Scientific and Practical Center, Enthusiasts Sh. 86, 111123 Moscow, Russia

**Keywords:** gastrointestinal stromal tumors (GISTs), novel *KIT/PDGFRA* mutations, “wild type” (WT) GISTs, the overall survival (OS), *BRAF* mutations, SDHB deficiency

## Abstract

Background/Objectives: Gastrointestinal stromal tumors (GISTs) are the most common malignant mesenchymal tumors that compose a heterogeneous group due to various molecular alterations. This study aimed to determine the molecular characteristics of patients with GIST treated between 2002 and 2017 and to follow their overall survival (OS) over 20 years. Methods: DNA was isolated from surgical biopsies of 490 patients with gut sarcomas and was analyzed using PCR and Sanger sequencing of hotspots of *KIT*, *PDGFRA* and *BRAF* genes. Immunohistochemical (IHC) staining was used to determine SDHB expression. Results: 417 GIST patients, including 13 children, were included; females predominated (male-to-female ratio 0.65), median age of 55.4 years old. *KIT* and *PDGFRA* mutations were found in 288 GISTs (69.1%) and 52 (12.5%), respectively. A total of 25 *KIT* and 3 novel *PDGFRA* mutations were revealed in 30 GISTs. *KIT/PDGFRA* mutations were not detected in 77 (18.5%) GISTs; among them were 2 NF1 GISTs, 10 with *BRAF* mutation, 14 with SDHB deficiency, and 3 “pediatric” GISTs. The overall survival (OS) of 280 GIST patients over more than 20 years. Genetic changes have a certain prognostic value in addition to such factors as tumor size, mitotic index, and location. The OS of GIST patients with *KIT* mutations was lower than that of patients with *PDGFRA* mutation, *BRAF* mutation, or SDHB deficiency, but the difference was non-significant. Conclusions: Our study highlights the heterogeneous nature and molecular features of GISTs in Russian population. The precise molecular characterization of the tumor should be considered an integral part of modern personalized GIST treatment.

## Simple Summary

Gastrointestinal stromal tumors (GISTs) are the most common malignant mesenchymal tumors classified as soft tissue sarcomas and accounting for 2% of all gastrointestinal malignancies. GISTs are heterogeneous group of tumors that includes a variety of molecular subtypes that have different natural histories and treatment. The aim of the study was to determine the spectrum and frequency of genetic alterations in a large cohort of Russian patients with GIST, who received treatment between 2002 and 2017, paying attention to adult and young patients. One of the goals of the study was to obtain data on the overall 20-year survival rate of patients with various genetic changes.

## 1. Introduction

Gastrointestinal stromal tumors (GISTs) are the most common malignant mesenchymal tumors classified as soft tissue sarcomas and accounting for 2% of all gastrointestinal malignancies [1]. The global incidence of GISTs varies between 4–22 patients per million individuals per year, with variations by region and over time, with an annual incidence of 10–12 cases per million in Western countries [2]. GIST can appear anywhere in the digestive tract: they are most frequent in the stomach (55–60%) and small intestine (30–35%), compared to the esophagus (<1%), colorectal (5%) or extra-gastrointestinal stromal tumors (EGISTs, 5%) [1,3,4]. Most patients with GIST are 60–65 years old, and the gender ratio of patients (male/female) ranges from 0.94 to 1.20 in different studies [2,3,4,5].

GISTs are a heterogeneous group of tumors that includes a variety of molecular subtypes that have different natural histories and treatments. Most GISTs originate from interstitial cells of Cajal (ICC), pacemaker and neuromodulator cells in the GI tract [1,3,5,6]. The most characteristic feature of GISTs that allows them to be distinguished from other mesenchymal gastrointestinal tumors is the expression of the CD117 antigen, the transmembrane tyrosine kinase KIT, a receptor for stem cells. KIT is normally expressed in Cajal cells in the gastrointestinal tract and plays an important role in developing the gut’s normal pacemaker system. The pathological diagnosis of GIST relies on cellular morphology and immunohistochemistry (IHC), where CD117 (KIT) is stained positive in over 90% of cases [1,5,6]. In 1998, Hirota and colleagues discovered that activating KIT mutations lead to a permanently active protein kinase, driving the major mechanism of GIST oncogenesis [7]. A smaller proportion of GISTs develop from telocytes, a distinct type of fibroblast-like cells that are another type of interstitial cells of the gut wall and are PDGFRA-positive [8]. PDGFRA is homologous to KIT and represent receptor tyrosine kinase for the platelet growth factor alpha [3].

Molecular studies have shown that 85–90% of GISTs possess mutually exclusive gain-of-function mutations, either in *KIT* (70%) or *PDGFRA* (10–15%) genes [1,3,4,5]. Both mutated KIT and PDGFRA lead to the pathogenesis of GIST through the activation of downstream signaling pathways, including RAF-MEK-MAPK, PI3K/AKT/mTOR and JAK-STAT [1,3,5]. It was shown that KIT and later PDGFRA became the targets for the tyrosine kinase inhibitor (TKI) imatinib [9,10]. Response to imatinib depends on the type and location of the mutation. Imatinib has become the standard first-line treatment for patients with unresectable or metastatic GISTs [10,11,12,13]. However, targeted therapy eventually leads to the development of drug resistance. Secondary mutations play a major role in this process, allowing for the selection of cells that are resistant to the treatment. Several other TKIs (sunitinib, regorafenib, repritinib, aprotinib, and others) were applied, and today five lines of targeted therapy are used based on the mutation status of GISTs [5,14,15].

The remaining 10–15% of tumors, formerly known as *KIT/PDGFRA* WT GISTs (“wild type” GISTs), may have an aberration in one of several genes, coding for BRAF, NF1, RAS, or SDH complex [3,4,5,14,16,17]. Mutations in serine-threonine kinase BRAF, neurofibromin NF1, and GTPase RAS induce activation of the RAF-MEK-MAPK signaling pathway [5,14]. It is assumed that BRAF-mutated GISTs can derive from smooth muscle cells [18].

GISTs with KIT/PDGFRA or BRAF, NF1, RAS mutations are SDH-competent, while 5% of GISTs are SDH-deficient and arise from mutations or epigenetic changes affecting the SDH (succinate dehydrogenase) complex, which consists of four A/B/C/D subunits. Loss-of-function of SDH leads to accumulation of succinate that induces increased transcription of HIF1-alpha-related genes and proteins, resulting in decreased demethylation. Some SDH-deficient GIST patients have mutations in A/B/C/D subunits of SDH, while other GISTs exhibit epigenetic silencing (hypermethylation) of SDHC [14,19,20].

GIST patients in whom sequencing fails to demonstrate mutation in *KIT/PDGFRA* or *BRAF* and who have a competent SDH complex may have driver alterations in other genes. Translocations of *NTRK, FGFR*, and *ALK* (fusion genes) were detected in rare GIST cases using NGS (next-generation sequencing) [5,14,17]. NGS allows detection of rare mutations in *TP53, CREBBP, CDKN2A, CHEK2,* and other genes in addition to driver genes [14,17].

Molecular analysis of GISTs is of great biological and clinical significance. The full understanding of *KIT* and *PDGFRA* as GIST oncogenic drivers, together with the remarkable success of tyrosine kinase inhibitors (TKIs), has significantly improved overall survival in these patients. The response to imatinib, sunitinib, and other TKIs and survival of patients depend upon specific mutations in GISTs [3,4,5,14,15].

The aim of this work was a molecular characteristic of the cohort of 417 patients with GISTs treated at N.N. Blokhin Cancer Research Center in 2002–2017. We conducted this retrospective analysis to provide much-needed insights into the genetic makeup and clinicopathological characteristics of patients in the Russian population. Part of the data on the presented cohort has been published earlier [21,22,23,24,25,26]. One of the goals of the study was to obtain data on the overall 20-year survival rate of GIST patients with various genetic alterations.

## 2. Materials and Methods

### 2.1. Materials

In this manuscript, we present results from a study of 417 GIST patients: 330 were operated on and treated at the N.N. Blokhin Cancer Research Center, while 87 were operated on at other hospitals and then admitted to the center for diagnosis and subsequent treatment. A total of 490 patients with different gut sarcomas (leiomyosarcoma, leiomyoma, schwannoma, neurofibroma, fibrosarcoma, synovial sarcoma, angiosarcoma, desmoid, desmoplastic sarcoma, fibrosis polyps) were tested, and as a result, 417 patients were diagnosed with GIST. We collected information on GIST patients’ gender, age, primary tumor site, tumor diameter and mitotic activity, histological and immunohistochemical (IHC) characteristics, *KIT* and *PDGFRA* pathogenic variants, response to targeted therapy, and overall survival. The diagnosis of GIST was made based on histological, immunohistochemical, and genetic analysis of the sections from paraffin-embedded surgical biopsies, which were examined by a specialized pathologist (O.A.A.). The DNA samples were obtained from the tumor tissue microdissections of these sections. The relevant patients with diagnosis of GIST had to display tumor morphology corresponding to GIST and positive immunostaining for KIT (CD117) protein, CD34, and frequently expressed tumor markers (smooth muscle actin, desmin, S100, and other proteins) [21,23]. The mitotic index was evaluated per 50 high-power fields (HPF) by examination of hematoxylin and eosin staining. The IHC staining of the succinate dehydrogenase subunit B (SDHB) expression was analyzed in the sections of WT GIST patients with rabbit antibodies (clone EP288, AC-0256RUOC, Epitomics Inc., Burlingame, CA, USA, 1:2000) and Dako EnVision Flex (Agilent Technologies, Glostrup, Denmark) detection system by a specialized pathologist (N.A.K.) [25].

### 2.2. Clinical Genomic Sequencing

All tumor samples were examined for the presence of somatic mutations in GIST actionable genes [22,24,25,26]. A total of 523 DNA samples from 490 patients were tested. Genomic DNA extraction was performed after manual dissection under microscopic guidance from FFPE (formalin-fixed and paraffin-embedded) tumor sections containing at least 80% of tumor cells. Genomic DNA was isolated from the tumor cells collected from tissue sections using standard protocols and was purified using the QIAamp DNA FEPE Tissue kit (Qiagen Inc., Valencia, CA, USA), and the DNA quality was assessed for each specimen. All GISTs were centrally examined for somatic mutations in *KIT* exon 11 by polymerase chain reaction amplification and then for mutations in exons 9, 13, and 17 of *KIT*, in exons 12, 14, and 18 of *PDGFRA*, and in exon 15 of *BRAF*. The nested polymerase chain reaction (nested-PCR) was developed to increase the sensitivity and specificity of PCR in cases where PCR did not reveal the product. The nested-PCR involved two consecutive amplification reactions using different primer pairs that were designed by I.S.B. (Supplementary Table S1). The PCR product from the first amplification reaction served as the template for the second PCR round, which was primed with oligonucleotides located within the first primer pair. Using two primer pairs allows more cycles, increasing PCR sensitivity. The reaction’s improved specificity derives from the binding of two separate primer sets to the same target template. PCR products were analyzed with direct right and reverse Sanger sequencing using an automated fluorescence-based cycle sequencer (ABIPRISM 3130; Life Technologies—Thermo Fisher Scientific, Waltham, MA, USA) [22]. However, the latest GIST samples were analyzed with a sequencing reaction that was performed according to the manufacturer’s protocol using the BigDye^®^ Terminator v3.1 Cycle Sequencing Kit (Thermo Fisher Scientific, USA) [24]. Sequencing was performed using an ABI Prism 3730 capillary sequencer (Applied Biosystems, Waltham, MA, USA). The results were analyzed using the Vector NTI Advance 11.1 program (Life Technologies, Waltham, MA, USA).

The classification of the gene variants was performed and supported by consulting the database “Catalogue of Somatic Mutations in Cancer” (COSMIC; https://cancer.sanger.ac.uk/cosmic) and the database “Clinically relevant variation” (ClinVar; www.ncbi.nlm.nih.gov/clinvar) in June 2026.

### 2.3. Statistics and Survival Analyses

The statistical processing of the research results was performed using the application software package Statistica v 10.0 for Windows (Dell, Round Rock, TX, USA), and GraphPad Prism 10.3.1 for Windows (GraphPad Software, Inc., San Diego, CA, USA), which include the Kaplan–Meier test, log-rank test (Mantel–Cox), and Gehan–Breslow–Wilcoxon test (chi-square). We assumed a critical significance level of 0.05 for statistical hypothesis testing. Time-to-event data were summarized using Kaplan–Meier methodology with associated 2-sided 95% CIs. *p*-values were nominal, per the testing design.

The overall survival rate was monitored until 2025 in 280 patients treated at the N.N. Blokhin Cancer Research Center. Overall survival (OS) was calculated from the date of first histologic diagnosis (surgical pathology) until patient death.

## 3. Results

### 3.1. Clinical Characteristics of the GIST Cohort

Immunohistochemical (IHC) and molecular analyses of 490 patients with mesenchymal tumors of the digestive tract revealed 417 patients with GISTs, of which 404 were adult patients, aged 21–84 years old (median age at diagnosis was 55.4 years old), Table 1. There was a predominance of female patients: 164 men and 253 women, with a male-to-female ratio of 0.65. Thirteen patients (3.1%) were children (9–20 years old), including 2 boys and 11 girls, with a median age of 15.6 years old.

The majority of tumors were of gastric origin (*n* = 220, 52.8%), followed by the small intestine (*n* = 145, 34.8%), including 26 (6.2%) duodenal tumors. Colorectal tumors were found in 22 (5.3%) patients. These tumors were localized in the colon (8) and rectum (14), while 2 tumors were found in the esophagus (0.5%). Extra-gastrointestinal stromal tumors (EGISTs) were identified in 24 (5.8%) patients. Four patients had metastatic tumors with unknown localization of the primary GIST.

Histopathological evaluation showed that 63.6% of tumors had spindle cell morphology, 16.1% had the epithelioid subtype, and 20.3% had the mixed epithelioid/spindle cell subtype. IHC analysis of CD117 and CD34 expression was performed on 362 and 309 patients, respectively. CD117 positivity was revealed in 95.6% of the tested patients, and 77.7% were CD34 positive. Tumor size and mitotic index were available in 308 and 349 GISTs, respectively, and were categorized according to various risk classifications.

### 3.2. Genomic Molecular Testing

#### 3.2.1. The Genomics of GISTs with KIT Mutations

All GISTs (*n* = 417) were examined for somatic mutations in the *KIT* and *PDGFRA* genes using PCR amplification and direct and reverse Sanger sequencing. *KIT* mutations were found in 288 GISTs (69.1%): in 286 (70.8%) adult patients and in two children (15.4%). *PDGFRA* mutations were found in 52 exclusively adult patients (12.5%). Meanwhile, Sanger sequencing found no *KIT/PDGFRA* mutations in 77 (18.5%) GISTs.

*KIT* mutations were more frequent in the spindle-cell GISTs (194 from 220), while *PDGFRA* mutations dominated in epithelioid and mixed cells (36 from 52). *KIT* mutations are significantly more common in spindle-cell tumors, while the epithelioid type is characteristic of GIST with *PDGFRA* mutations (*p* < 0.001, χ2 = 9.488, df = 4). Clinico-pathological characteristics are presented in Table 2. *KIT* mutations were detected in 2 cases in esophagus, 59.1% (130 from 220) gastric tumors, 77.2% (112 from 145) intestinal GISTs, 90.9% (20 from 22) colorectal, 66.7% (16 from 24) EGISTs and in 2 from 4 tumors with unknown primary location (Table 2).

Mutations in *KIT* exon 11 were found in 245 (58.8%) cases, in exon 9—in 34 (8.1%), in exons 13 and 17—in 5 (1.2%) and 4 (1.0%) cases, respectively (Figure 1). Several GISTs had two simultaneous mutations in exon 11 and both in exon 11 and exon 13 of the *KIT* gene.

We identified the following mutations in *KIT* exon 11, affecting the cytosolic JM domain: 127 deletions (del), 70 substitutions, 30 deletions/insertions (delins), 15 duplications, and 3 insertions. There were identified 28 novel mutations (25 *KIT* and 3 *PDGFRA*) in 30 GISTs, with two mutations identified in two tumors of different locations (Table 3). These mutations were absent in COSMIC and ClinVar.

All *KIT* mutations are located in the open reading frame, except for two frameshift mutations that were identified in the intestinal GISTs: c.1755Cdel* (p.P585del) (Figure 2a) in a 62-year-old male patient, and c.1703–1727delins (p.Y568_576 delins CVGS*), (Figure 2b) in a 9-year-old boy. These mutations were not reported previously (new, marked with*).

*KIT* exon 11 mutations were heterogeneous in the classic hotspot region at the 5′- end of the exon (codons 550–561). There were 22 GISTs with mutations in exon 11 that started in intron 10 or/and had 19 deletions and 3 delins (deletions with insertions) that included codons 550–556 (upstream codon 557). The most common *KIT* mutation p.W557_K558del, was identified in 26 cases (10% of *KIT* exon 11 mutations), and 4 tumors contained various p.W557_K558delins. Novel mutation p.W557_K558delins CE* (c.1671–1672GA/TG) was identified in two GIST cases in the stomach and colon (Figure 2c). Genomic analysis revealed 53 GISTs with deletions and 18 GISTs with delins spanning both or one of codons 557/558. Among the mutation sites downstream of codon 558, we found 30 deletions and 5 delins located in the distal part of *KIT* exon 11. Some deletions involve tyrosine residues p.Y568 and p.Y570, which have significant, specific effects on KIT signaling pathways, as phosphorylation of Tyr568 and Tyr570 plays an important role in KIT activation [27].

Single non-synonymous substitutions in *KIT* exon 11 were detected in 70 GIST samples. Substitutions affecting codons 557, 559, 560, and 576 were frequent: p.V560D and p.V560G were found in 22 and 4 tumors, respectively; p.V559D, p.V559A, and p.V559G were found in 14, 6, and 5 tumors, respectively; and p.W557R was found in 9 tumors. Six tumors harbor the p.L576P mutation, and one GIST had the rare substitution p.L576F. Novel mutations p.Y553D* (c.1657T>G) (Figure 2d) and p.E554D* (c.1662A>T), (Figure 2e) were found, as well as rare p.K558P and homozygous p.N567D substitutions. Simultaneous mutations p.L576F and homozygous p.M552_W557del were found in the intestinal tumor in a 48-year-old man. Two simultaneous KIT substitutions p.K550N and p.K558P, were found in GIST in a 68-year-old man.

There were 15 duplications and 3 insertions in *KIT* exon 11 revealed in GIST patients under study. Women prevailed among them (*n* = 12), and their average age was 61.7 years old (range 43–76 years old), while the average age of men (*n* = 6) was 53.8 years old (range 43–64 years old). Tumors were located in the stomach (*n* = 14), esophagus (*n* = 1), intestine (*n* = 2), and colon (*n* = 1). Most GISTs were spindle cell, large (>10 cm), with high mitotic activity (>5 in 50 HPF x400), yet these patients had good survival (median overall survival was 74.3 months). All duplication/insertions affected codons 570–590. Several mutations were reported earlier: p.I571_D579dupl (*n* = 2), p.I571_H580dupl (*n* = 2), and p.T574_P585dupl (*n* = 2), but most duplications/insertions were identified for the first time. For example: p.P573_L576dupl (c.1719–1728dupl, Figure 2f), p.Q575_P577dupl (c.1723–1731dupl, Figure 2g), p.P577ins PF (c.1730insATTTCC, Figure 2h), p.Y578_D579dupl (c.1732–1737dupl. Figure 2i).

All mutations in exon 9, coding the extracellular KIT domain, were duplications p.A502_Y503, identified in 34 (8.1%) tumors. These mutations were more common in the small intestine (*n* = 29, 85.3%) as previously reported [28], but were also found in two gastric, rectal, and two EGIST samples.

Mutations in exon 13, which encodes the KIT TK1 domain, were detected in 5 (1.2%) cases. Substitutions p.K642E, p.K642Q, and rare p.T632A were found in the stomach, while rare p.Y646C and p.Y646H mutations were detected in two EGISTs. Gastric tumors with the substitutions in exon 13 of *KIT* showed increased invasiveness, which is consistent with previously reported data [29]. Two simultaneous mutations, p.L576P in exon 11 and primary p.Y646C in exon 13, were detected in EGIST in a 44-year-old woman.

Primary mutations in *KIT* exon 17, coding the TK2 domain, were found in three gastric tumors: p.D816V, p.D820V, and rare p.T801N. Two simultaneous KIT substitutions were found in the intestinal GIST: a novel p.N819Y (c.2457A>T)* and homozygous p.N822K (Figure 2j). Taken together, primary *KIT* mutations in exons 13 and 17 were more common in the stomach (6/9 GISTs), which does not align with data showing these mutations are more common in the intestine [29]. The most GISTs with *KIT* mutations in exons 13 or 17 had spindle-cell histology, except for an epithelioid gastric tumor with a rare p.T801N KIT mutation in a 16-year-old girl.

We also tested secondary DNA mutations in *KIT* exons 13 and 17 in DNA samples isolated from GISTs that recurred or metastasized after imatinib therapy. Analysis of 18 GISTs with primary mutations in exon 11 (*n* = 17) and exon 9 (*n* = 1) revealed secondary mutations in 9 (50%) GISTs in exon 17: p.D816H, p.D820H (*n* = 3), p.D820E, p.D820Y, p.S821C, p.N822K, p.Y823D. Thus, the most common mutations associated with secondary resistance were localized in exon 17 of *KIT*.

#### 3.2.2. GISTs with PDGFRA Mutations

*PDGFRA* mutations were found in only 52 adult patients (12.5%), mainly in gastric tumors (*n* = 42, 80.8%). Mutations in exon 18 of the *PDGFRA* gene were found in 46 (11.0%) tumors. The most common *PDGFRA* mutation, p.D842V, which is related to GIST resistance to imatinib, was found in 26 stomach tumors (6.2%) or in 50% of all *PDGFRA* mutant GISTs, which is lower than usually found (70%) [3,5]. Non-p.D842V mutations include substitutions and deletions, were common among our GIST patients with mutations in *PDGFRA* exon 18. Five substitutions were found in tumors in the stomach: p.I843V, p.S847L, p.Y849N (*n* = 2), and novel p.A821S (c.2461G>T)* (Figure 3a), while p.S847L was also found in the intestine. Deletions in *PDGFRA* exon 18 were found in 14 GISTs, including 9 female patients (64%). The most frequent deletions were p.I843_D846del (*n* = 3), p.I843_S847delinsT (*n* = 2), p.D842_H845del. Novel mutation p.C835del (c.2503–2505del)* was found in two tumors localized in the stomach and in the intestine (Figure 3b).

One patient, a 48-year-old female, had rare mutation in exon 14 of *PDGFRA:* p.A603V (c.1808 C>T) in the rectal GIST (Figure 3c). Five GISTs (1.2%) had mutations in *PDGFRA* exon 12, which encodes the JM protein domain: p.R558C was found in 4 tumors localized in the stomach, intestine, colon, and EGIST, while the novel mutation p.E563V* (c.1697A>T) (Figure 3d) was found in the stomach.

#### 3.2.3. *KIT/PDGFRA* “Wild-Type” GISTs

Genetic analysis of *KIT* and *PDGFRA* mutations, using Sanger sequencing, did not reveal mutations in 77 (18.5%) patients with *KIT/PDGFRA WT,* or “wild type” GIST. These tumors were CD117-positive and were characterized by the typical histological and immunohistochemical features of GISTs [23,25]. In total, 77 patients had *KIT/PDGFRA WT* GISTs, women predominated (*n* = 53), median age 45-year-old, and 24 men, median age 53.6-year-old. Of these, 66 GISTs (16.3%) occurred in 404 adult patients, while 11 tumors were detected in children (11/13; 84.6%). “Wild-type” GISTs were localized in the stomach (*n* = 42), small intestine (*n* = 30), EGISTs (*n* = 4), and one patient had “wild type” GIST with an unknown primary site.

A group of *KIT/PDGFRA* WT GISTs is heterogeneous and composed of several distinct entities with multiple unique molecular alterations. Ten patients with the BRAF p.V600E mutation were identified, which account for 13.0% of “wild type” GIST or 2.4% of all the tumor samples (*n* = 417). Most patients with BRAF mutations were women (9/10), with an average age of 45.0 years (12–76 years); two were young girls. Most tumors (8 out of 10) were localized in the stomach and two—in the small intestine, which does not coincide with the data that GISTs with BRAF mutations are mainly localized in the small intestine [30].

Two “wild type” GIST patients, 51-year-old man and a 55-year-old woman, had multiple tumors in the duodenum. These GISTs had clinical features of neurofibromatosis (NF-1-associated GISTs), as they displayed subcutaneous neurofibromas. The NF-1-associated GISTs were spindle-cell CD117-positive tumors, aggressive and resistant to imatinib. These two GISTs had not undergone genetic testing, as both patients displayed classic NF-1 phenotypic features.

All GISTs with *KIT*, *PDGFRA*, *BRAF*, or *NF-1* mutations are SDH-competent [5,14]. 5% of GISTs are “wild type” SDH-deficient tumors that compose the unique class of GIST with distinct clinical, morphological, and immunohistochemical features which distinguish them from other GISTs [14,20,31]. The SDH-deficient GISTs are defined by loss of IHC SDHB expression, as loss of SDHB expression becomes negative whenever there is biallelic inactivation of any SDH component. Therefore, loss of SDHB expression serves as a marker for syndromic disease, usually due to a germline mutation in one of the SDH subunits [32]. We examined SDHB expression in 20 patients with “wild type” GISTs for whom paraffin-embedded tissues were available, and 13 of them showed loss of SDHB expression [25]. Among SDHB-deficient patients, women predominated (*n* = 11) and one woman had Carney-Stratakis syndrome, while three women had an incomplete Carney triad (GIST and chondroma). In addition to these patients, another 49-year-old woman was diagnosed with Carney’s triad, but this archived histologic material was not available for SDHB analysis. Thus, the study involved 14 patients (3.4%) with SDHB deficiency, with a predominance of women (12 patients, 85.7%), with an average age of 39.2 years old. All SDHB-negative tumors were localized in the stomach; four patients suffered from multicentric lesions, and more than half of the patients had distant metastases [25,26]. In addition, three girls aged 15 to 16 years old had “pediatric GISTs”, which are epithelioid tumors of the stomach with a characteristic clinical picture and SDH deficiency, but their tumor samples were not available for SDHB analysis. Thus, we suppose that 17 patients may have SDH deficiency. We also included patients with *KIT/PDGFRA/BRAF WT* GISTs, which should be analyzed by next-generation sequencing. This will need to be done in future experiments.

#### 3.2.4. The Overall Survival Rates of GIST Patients

All patients with GIST who participated in this study received surgical treatment, and most of them received imatinib as first-line targeted therapy, except patients who were operated on and died before 2004. We retrospectively tracked the overall survival of 280 of 417 GIST patients treated in N.N. Blokhin Cancer Research Center for more than 20 years (Table 4). The clinical characteristics of the 417 (Table 1) and 280 (Table 4) patients match well. Therefore, overall survival analysis of 280 patients reflects the overall picture. We were unable to follow up 137 patients due to missing survival data. Data for the 137 patients not included in the study are presented in Supplementary Table S2.

It is well known that the survival of GIST patients depends on tumor size, mitotic index, and tumor location, which are prognostic factors [1,5,33]. Our results support these findings, as patient survival differed significantly by tumor size (*p* < 0.0001) and mitotic index (*p* = 0.0006). However, a non-significant trend in differences was observed in the OS of patients with different GIST locations (*p =* 0.069, but *p* = 0.032 for trend) (Figure 4).

Thus, overall survival was best in patients with GIST in the stomach (Me 175 months) and colorectal GISTs (Me 142 months), followed by patients with intestinal GISTs (Me 124 months). This contradicts the literature, which shows that patients with colorectal GIST have quite low survival rates [33]. Patients with EGIST had the worst overall survival (Me 61 months). The median overall survival in patients with gastric GIST is significantly higher than in those with EGIST (p-log-rank (Mantel–Cox) test: *p* = 0.0175, χ2 = 7.953, df = 1; Gehan–Breslow–Wilcoxon test: *p* = 0.0048, χ2 = 5.651, df = 1).

Genetic changes also have a certain prognostic value. An analysis of survival rates of GIST patients based on gene mutations showed that the overall survival of patients with *KIT* mutation (Me 124 months) was lower than that of patients with *PDGFRA* mutation (Me not reached) or “wild-type” GISTs (Me 250 months) (Figure 5a). The overall survival of GIST patients with mutations in exon 11 of *KIT* was higher (Me 132 months) than that with mutations in exon 9 (Me 67 months) or exons 13/17 (Me 52 months) (Figure 5b). GIST patients with deletions in exon 11 of *KIT* revealed shorter survival (Me 117 months) than patients with substitutions (Me 179 months) or duplication/insertions in exon 11 of *KIT* (Me not reached) (Figure 5c). GISTs with duplication/insertions in the distal part of exon 11 mainly originated in the stomach in women and had a relatively indolent clinical behavior. The results obtained indicate certain trends, but no statistically significant differences were observed.

It has been shown previously that GIST patients (especially with gastric tumors) with mutations in codons 557_558 of *KIT* had shorter progression-free survival compared to the patients with other *KIT* exon 11 deletions, in spite of the fact that GISTs with exon 11 mutations on codons 557_558 showed robust responses to first-line imatinib treatment [29,31,34]. We did observe a slight difference in overall survival between patients with gastric GIST who had a deletion p.W557_K558del in exon 11 of *KIT* and patients with other deletions, but the difference was non-significant (Figure 5d).

GIST patients with *PDGFRA* mutations had a relatively high overall survival (Figure 5e). Five patients had mutations in exon 12, one patient had a rare mutation in exon 14, and 46 patients had different mutations in exon 18 of *PDGFRA*. It is well known that GIST with mutation p.D842V and other substitutions in exon 18 of *PDGFRA* involving codon 842 are resistant to imatinib and other TKIs (sunitinib, regorafenib etc.) but are sensitive to repritinib and avapritinib [35]. However, our patients with the p.D842V included in this study did not receive avapritinib, as it was approved in 2020. Nevertheless, we compared overall survival in GIST patients with p.D842V versus non-D842V mutations. GIST patients with substitutions and deletions not affecting codon 842 were sensitive to imatinib and showed high OS (Figure 5e), which is consistent with the results of previous studies [36,37].

Two *KIT/PDGFRA* WT GISTs associated with NF1 were localized in the intestine and showed poor survival rates. GIST patients with *KIT/PDGFRA* WT, namely with the BRAFV600E (Me has not been reached) and SDH deficiency (Me has not been reached) showed relatively good survival rates (Figure 5f). Both types of genomic changes were predominantly localized in stomach tumors in women. The 10-year OS rate of this group of patients with SDH deficiency was 82% [25]. It should be emphasized that the differences presented are not strictly reliable.

## 4. Discussion

GIST is the most common and best-studied sarcoma of the gastrointestinal tract. Over more than twenty years, data have accumulated on the heterogeneity, features, and diversity of these tumors. Activating mutations in *KIT* or *PDGFRA* disrupt the normal autoinhibitory state of receptor tyrosine kinases, leading to the constitutive, ligand-independent activation of MAPK, JAK/STAT3, and PI3K/Akt/mTOR downstream pathways, ultimately resulting in increased cell proliferation. It was shown that there are ethnic differences: the frequency of GIST is higher among African Americans, as well as Asians, than among Caucasians [38]. The frequency of *KIT* and *PDGFRA* mutations differs between patients of European and Asian origin, with *PDGFRA* mutations being more common in Europeans than in Asian patients, while mutations in exons 9, 13, and 17 of *KIT* are less common in Europeans than in Asians [36,39,40].

We were interested in the molecular profile of GIST in Russian patients. In 2024, about 2000 new GIST cases were registered, so the incidence rate among the Russian population was about 13 cases per 1 million people [41]. Previously, we conducted studies on the morphology [21,22,23,25], mutant status [22,24,25,26], and survival rates in small groups of patients with GIST (*n* = 120 [24] and *n* = 244 [26]). Here, we present the results of a retrospective study in which we analyzed 417 GIST cases from 404 adult patients and 13 children aged 9 to 20 years. The average age of adult patients was 55 years, while in Western countries the average age of patients with GIST is 60–65 years [1,2,3,4,5] or even 70–79 years [38]. Russian patients with GISTs were predominantly women, with a male-to-female ratio of 0.65, while most studies report male predominance or similar rates of incidence [1,2,3,4,5,38].

The most powerful predictor of targeted therapy efficacy for GIST is the mutational status of *KIT* and *PDGFRA* genes. We have identified primary *KIT/PDGFRA* mutations in 340 (81.5%) GIST samples using “nested PCR” and Sanger sequencing. All but two GISTs contained in-frame mutations but two tumors had frameshift *KIT* exon 11 mutations, that were not reported. It should be noted that we did not consider SNPs or silent mutations, like p.T632T in exon 13 of *KIT*, p.N570N in exon 12 of *PDGFRA*, or p.V824V in exon 18 of *PDGFRA*, etc.

Gastrointestinal stromal tumors are typically well-defined mesenchymal neoplasms and their molecular status has been studied intensively. Recently, accumulating evidence strongly indicated that the gene coding, epigenetic regulation, and pre- or post- protein translation of KIT mutants in GIST were quite different from those of wild-type (WT) KIT [42]. In addition to the typical *KIT* and *PDGFRA* gene mutations, rare primary *KIT* mutations have also been reported [43]. We identified 25 *KIT* and 3 *PDGFRA* mutations not previously reported, widening the spectrum of known mutations in GISTs. These mutations were identified in 30 GIST cases of different locations: stomach, small intestine, colon, esophagus, and EGIST. We identified two new mutations in GISTs from different locations: p.W557_K558delins CE in exon 11 of *KIT,* detected in the stomach and colon, and p.C895del in exon 18 of *PDGFRA,* found in the stomach and intestine. All novel mutations were heterozygous except for homozygous p.N822K in exon 17 of *KIT*.

It should be noted that many rare mutations were identified, such as p.T632A, p.K642Q, p.Y646C and p.Y646H in exon 13 of *KIT*, while the common mutation p.K642E was only found in one of five GISTs. Similarly, we detected the novel p.N819Y and the rare p.T801N in exon 17 of *KIT*. Mutations in exons 13/17 of *KIT* were prevalent in the gastric GISTs (6 of 9), which contradicts the finding that these mutations were more common in the intestine [29]. All GISTs with *KIT* mutations in exons 13/17 had the spindle-cell histologic features, except one in a young girl with the rare mutation p.T801N, and these tumors were aggressive, which is consistent with other data [29]. Overall survival was reduced in patients with mutations in exons 13/17 of *KIT*, despite most patients receiving sunitinib.

*PDGFRA* mutations were detected in 52 of 417 cases (12.5%) but only in adult patients with GIST (52 of 404, 12.9%). Of these, 46 (11.4%) GISTs had mutations in exon 18, including 26 (6.2%) cases with p.D842V, which were resistant to imatinib and other TKI (sunitinib, regorafenib). Patients with p.D842V are well known to be sensitive to avapritinib, repritinib, and crenolanib; however, our patients could not receive these drugs between 2002–2017. There were also 20 (4.8%) GIST patients with deletions and substitutions in exon 18 of *PDGFRA*, and their survival rates were quite high. The patients with mutations in exon 12 of *PDGFRA* demonstrated rather high overall survival.

In this study, 77 *KIT/PDGFRA* WT GISTs were identified, of which two were associated with NF-1(2.6%), and 10 had a BRAF mutation (13.0%), which activates the MAPK pathway, as well as at least 14 tumors had SDH deficiency (18.2%). Several patients with “wild-type” GIST were not tested for SDHB protein expression, as their blocks were not available for new IHC analysis. However, based on clinical features, age, and genetic analysis, it is possible that, in addition, three young girls with “pediatric” *KIT/PDGFRA* WT GISTs may have SDH deficiency.

Analysis of the overall survival rates has supported the findings that the survival of GIST patients depends on tumor size, mitotic index, and tumor location [1,3,5], and these differences were statistically significant. The best OS was observed in patients with GIST in the stomach, but patients with colorectal GIST also had a relatively good survival rate. This is in discrepancy with the literature data that show quite low survival rates in patients with colorectal tumors [36]. GIST patients with *KIT* mutations generally have worse survival outcomes compared to all GIST cases, though there is considerable heterogeneity. Patients with duplication-insertions in exon 11 of *KIT* had a better survival rate than other patients with *KIT* deletions or substitutions in exon 11 or with mutations in exons 9, 13, or 17. No significant differences in OS were found between patients with GIST with deletions in codons 557–558 and those with other deletions in *KIT* exon 11. However, we observed a trend: patients with GIST of the stomach with deletions at codons 557–558 had shorter overall survival, consistent with data [29,33,34].

GIST patients with PDGFRA mutations typically have better survival rates compared to all GIST cases [3,5,14], and we observe this trend in our patients. Many of them had GIST with deletions or substitutions encoded by exon 18 of *PDGFRA* other than p.D842V, and these tumors were sensitive to imatinib. Another group of patients had the p.D842V mutation, which is responsible for resistance to imatinib, and, despite the fact that they did not receive another targeted therapy, the overall survival data of patients with exon 18 mutations were quite high. Interestingly, patients with rare exon 12 *PDGFRA* mutations showed good OS, but there were few.

Patients with *KIT/PDGFRA WT* GIST with BRAF mutation or SDH deficiency showed more indolent clinical presentation and relatively good survival rates. Both types of genomic changes were predominantly localized in the stomach of women. Most our patients with BRAF mutation were women (9 of 10) with GIST in the stomach (8 of 10), while BRAF mutations were usually found in the intestinal tumors [5,14,30]. Patients with SDH deficiency were also predominantly women (12 of 14 with SDH-deficient GIST), as well as 3 girls with “pediatric” GIST in the stomach. These patients showed a 10-year overall survival rate of 82% [25]. Two *KIT/PDGFRA WT* GISTs associated with NF1 were localized in the intestine and showed poor OS. Several GISTs are *KIT/PDGFRA/BRAF/SDH* WT and next-generation sequencing should be used for analysis of the drivers.

This study represents the most detailed investigation of a large cohort of patients with GIST from Russia. This is the first work describing gastrointestinal tumors in children from Russia, including their clinicopathological status and mutations. For the first time, patients with *KIT/PDGFRA* WT GIST, their molecular profile, and the survival rates have been examined in the Russian population. Unfortunately, we could track survival for only 280 of 417 patients over 20 years. The OS of patients with various mutations was analyzed. In this study, we identified 25 novel *KIT* and 3 *PDGFRA* mutations. However, PCR and Sanger sequencing of gene “hotspots” cannot detect low-frequency mutant alleles (less than 20%) in the studied genes. The use of next-generation sequencing will make it possible to identify new driver genes and rare mutations.

## 5. Conclusions

In this retrospective study, we have tested the molecular profile of GISTs in Russian patients. This study was conducted using a large clinical dataset and focused on the specific characteristics and 20-year survival rates of a large group of patients. GISTs in Russian patients develop at an earlier age and are more common in women. For the first time, the molecular profile of GISTs in children from the Russian population was identified and discussed. Detailed genomic analysis revealed rare and new molecular variants. The spectrum of driver mutations we obtained is similar to that of European patients, but we identified some differences. A clear limitation of this study is our inability to use next-generation sequencing; we hope NGS will allow us to identify mutations we could not detect. Improving our understanding of the molecular mechanisms underlying the different GIST subtypes is needed to develop more effective therapeutic approaches. The precise molecular characterization of the tumor should be considered an integral part of modern personalized GIST treatment.

## Figures and Tables

**Figure 1:**
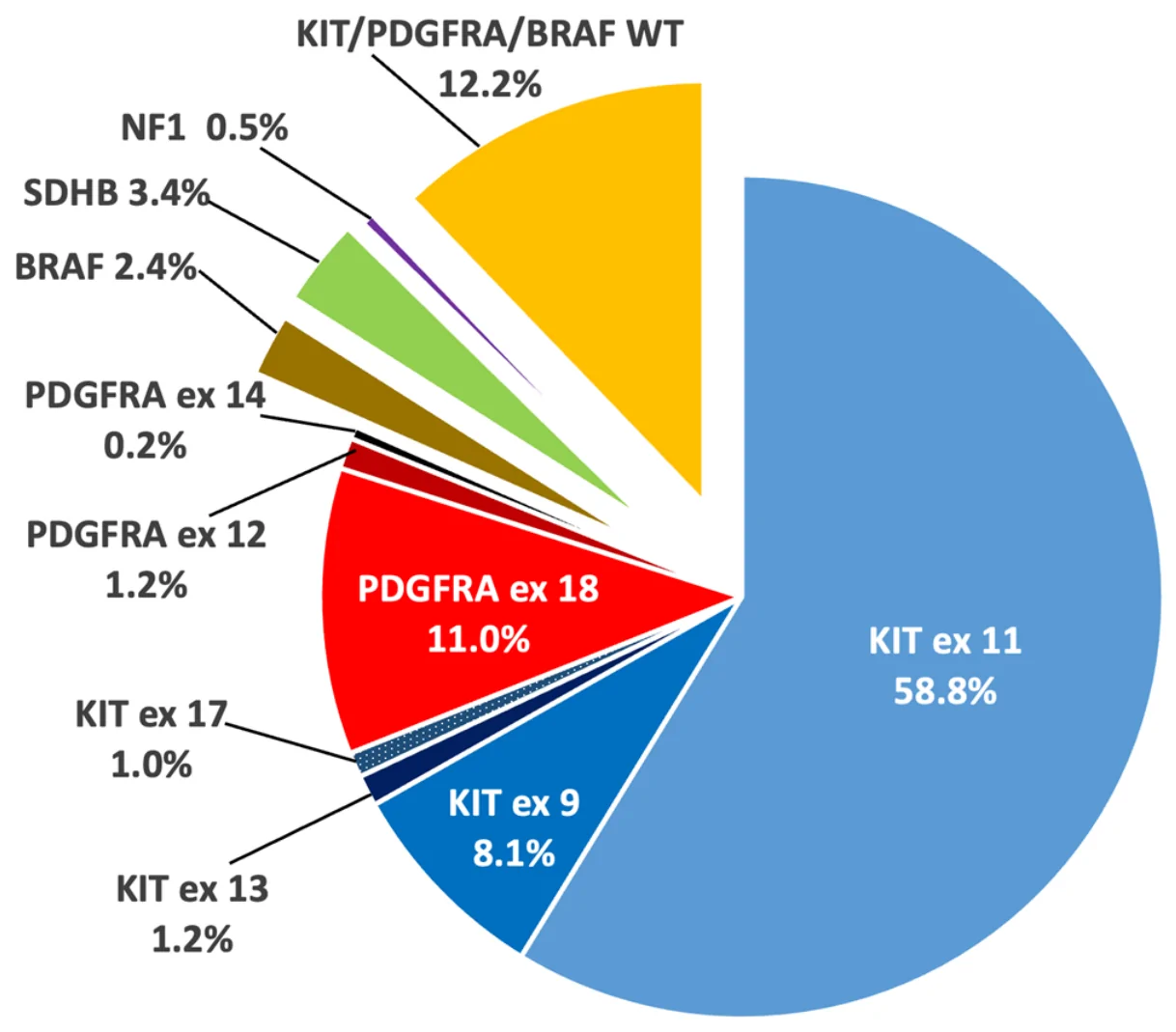
The frequencies of various genetic changes in 417 gastrointestinal stromal tumors in Russian patients.

**Figure 2:**
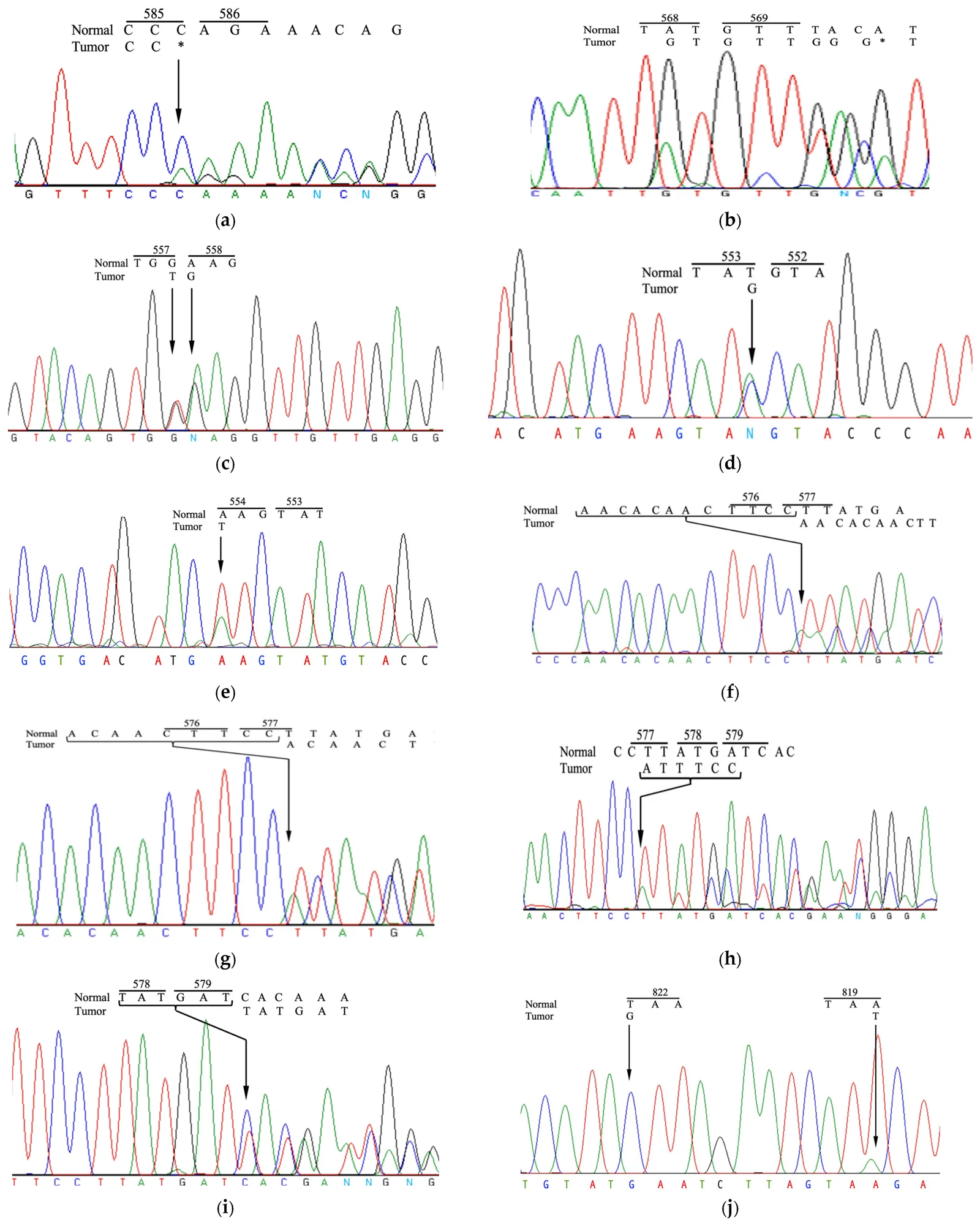
Novel mutations in *KIT* exon 11: (**a**–**c**,**f**–**i**)—sequencing in the right direction; (**d**,**e**,**j**)—reverse sequencing. (**a**) *KIT* ex 11 p.P585del, Frameshift (c.1755delC). (**b**) *KIT* ex11 p.Y568_576delins CVGS, Frameshift (c.1703–1727delins GTGTTGGGTC). (**c**) *KIT* ex11 p.W557_K558delins CE(c.1671–1672GA>TG). (**d**) *KIT* ex 11 p.Y553D (c.1657 T>G). (**e**) *KIT* ex 11 p.E554D (c.1662 A>T). (**f**) *KIT* ex 11 p.P573_L576dupl (c.1719–1728dupl). (**g**) *KIT* ex 11 p.Q575-P577dupl (c.1722–1730dupl). (**h**) *KIT* ex 11 p.P577ins PF (c.1730insATTTCC). (**i**) *KIT* ex 11 p.Y578_D579dupl (c.1732–1737dupl). (**j**) *KIT* ex 17 p.N819Y (c.2455A>T) + p.N822K homo (c.2466 T>G). * deleted nucleotide.

**Figure 3:**
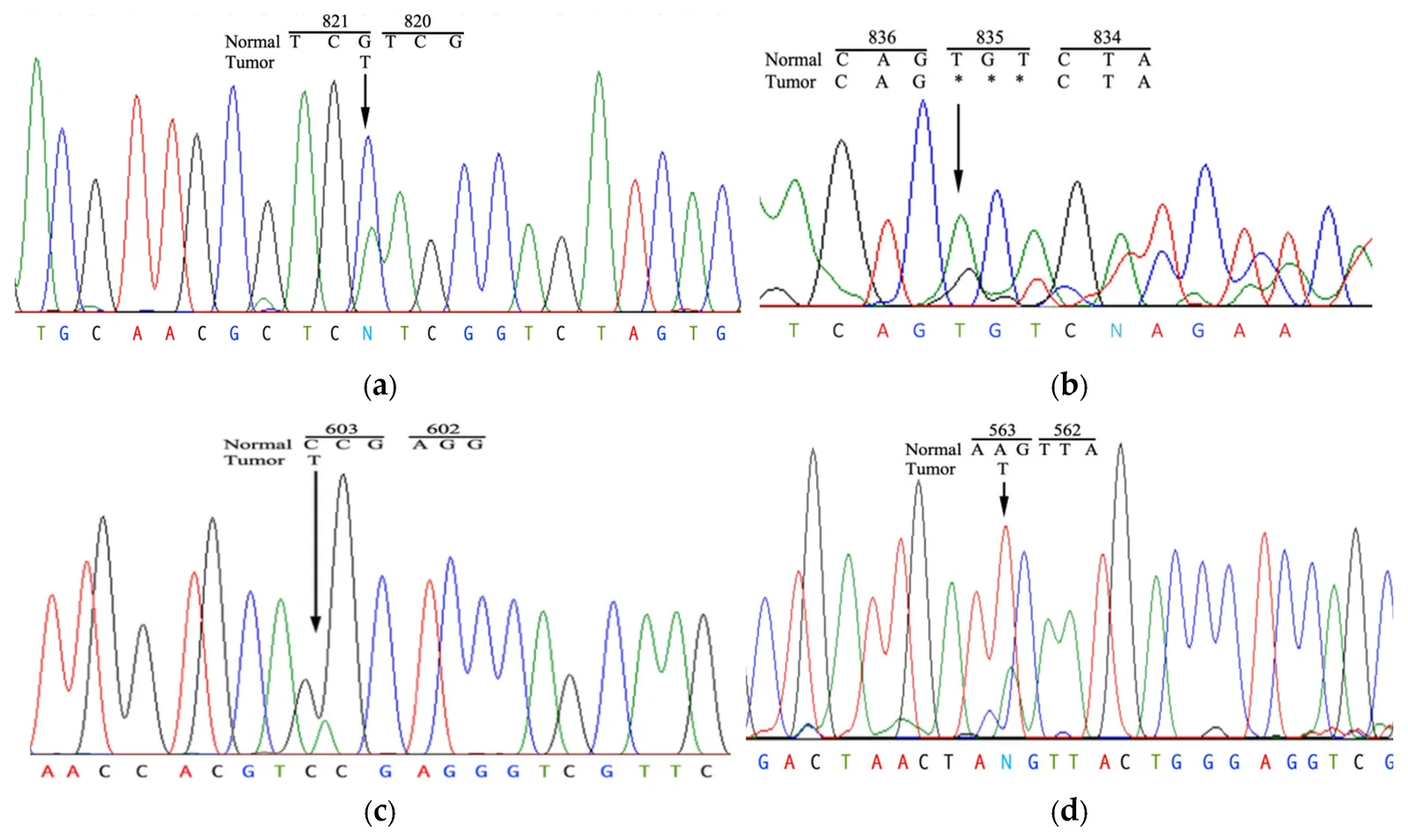
Mutations in *PDGFRA* exon 18 ((**a**,**b**) novel), in exon 14 ((**c**) rare) and in exon 12 ((**d**) novel), reverse sequencing. (**a**) *PDGFRA* ex.18 p.A821S (c.2461G>T). (**b**) *PDGFRA* ex.18 p.C835del (c.2503–2505 del). (**c**) *PDGFRA* ex.14 p.A603V (c.1808 C>T). (**d**) *PDGFRA* ex.12 p.E563V (c.1697A>T). * deleted nucleotide.

**Figure 4:**
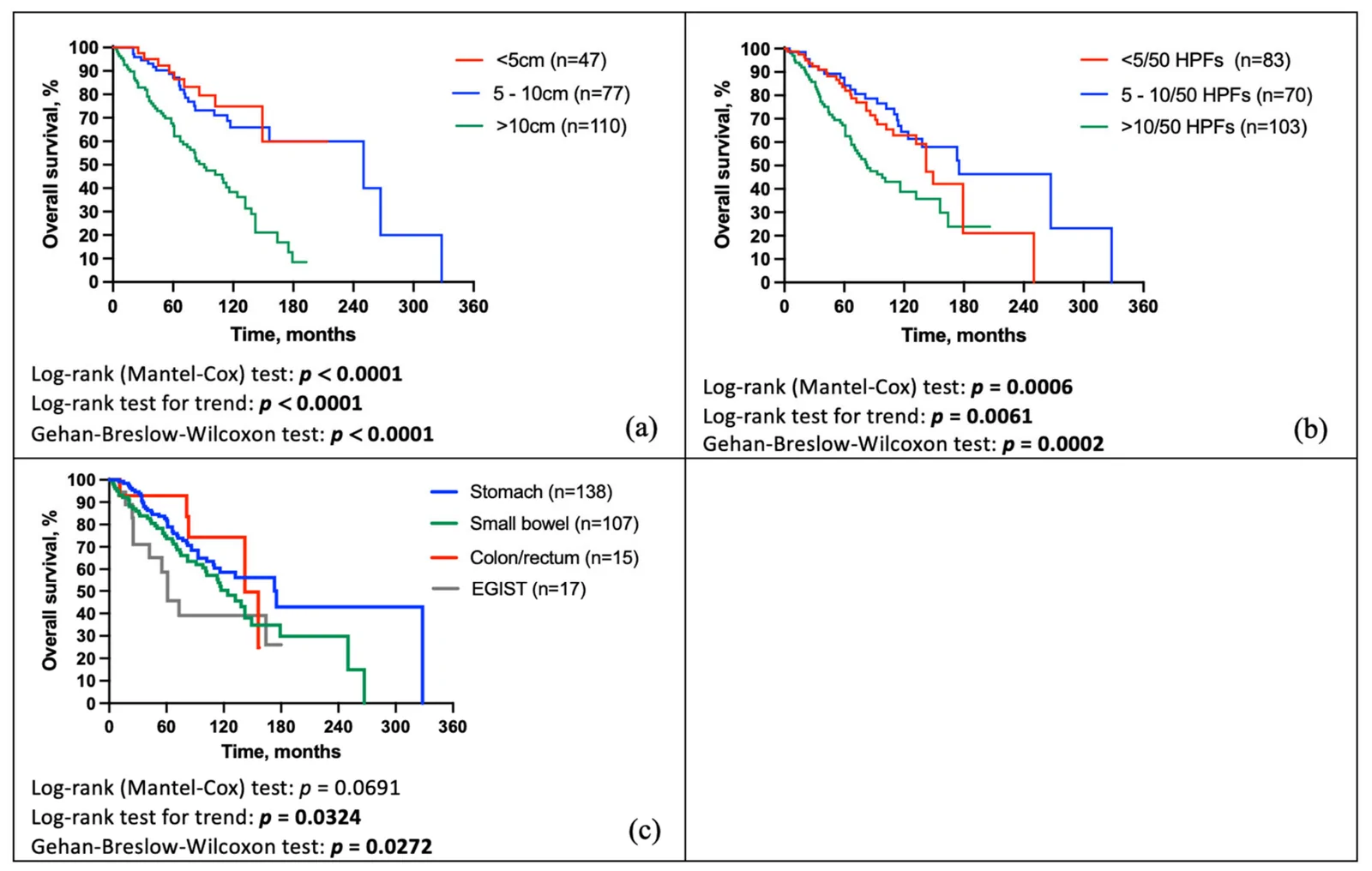
The overall survival of GIST patients depends on: (**a**) tumor size, (**b**) mitotic index, and (**c**) tumor location.

**Figure 5:**
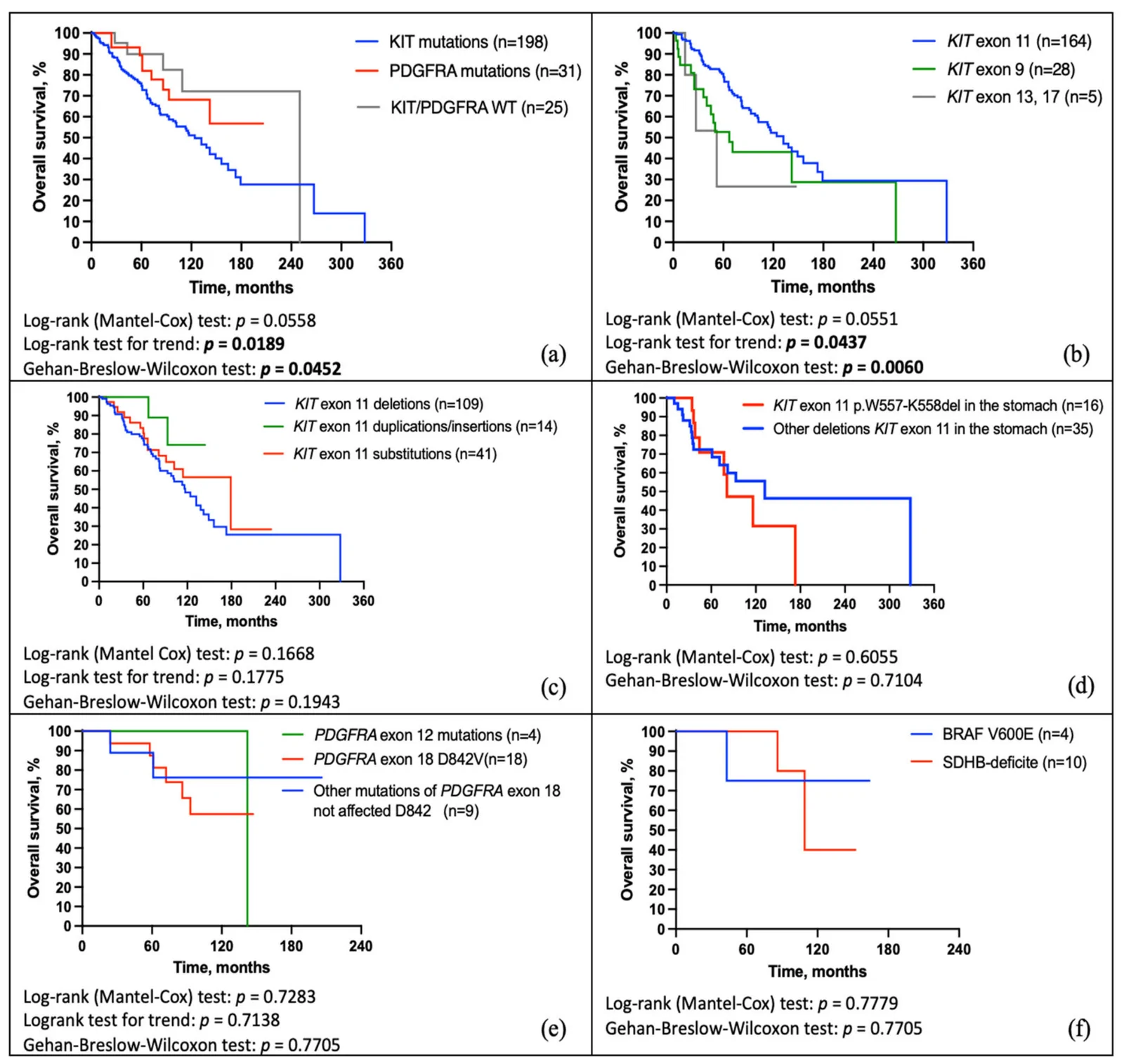
The overall survival rate of GIST patients depends on: (**a**) mutations in different genes, (**b**) mutations in different exons of *KIT*; (**c**) different types of mutation in *KIT* exon 11; (**d**) different deletions in *KIT* exon 11 in GIST of the stomach; (**e**) mutations in different exons of *PDGFRA*; (**f**) BRAF and SDHB alterations in GIST.

**Table 1:** The clinicomorphological characteristics of 417 patients with GISTs.

Characteristics	Number of Patients (%)
Sex	*n* = 417
Male	164 (39.3%)
Female	253 (60.7%)
Age at diagnosis	*n* = 417
Adult, (range, 21–84 years)	404 (96.9%)
median age 55,4 years	
Children (range, 9–20 years)	13 (3.1%)
median age 15,6 years	
Primary tumor location	*n* = 417
Esophagus	2 (0.5%)
Stomach	220 (52.8%)
Small intestine	145 (34.8%)
Rectum and colon	22 (5.3%)
Extra-gastrointestinal (EGIST)	24 (5.8%)
Unknown primary tumor	4 (1%)
Morphology	*n* = 390
Spindle cells	248 (63.6%)
Epithelioid cells	63 (16.1%)
Mixed cells	79 (20.3%)
CD117 expression	*n* = 362
Positive	346 (95.6%)
Negative	16 (4.4%)
CD34 expression	*n* = 309
Positive	240 (77.7%)
Negative	69 (22.3%)
Primary tumor size, cm	*n* = 308
0–5	75 (24.4%)
5.1–10	94 (30.5%)
>10	139 (45.1%)
Mitotic index, 50 HPF x400	*n* = 349
≤5	126 (36.1%)
5.1–10	91 (26.1%)
>10	132 (37.8%)

**Table 2:** Clinicopathological characteristics and mutational status of gastrointestinal stromal tumors.

GIST Cases	*N* (%) 417	KIT Mutations (69.0%) *n* = 288						PDGFRA Mutations (12.5%) *n* = 52				KIT/PDGFRA WT (18.5%) *n* = 77			
		Exon 9	Exon 11 (*n* = 245, 58.8%)			Exon 13	Exon 17	Exon 12	Exon 14	Exon 18					
			Deletions + Delins	Substitut.	Duplic. + Inser.	Substit.	Substit.	Substit.	Substit.	Subst.	Delet.	BRAF	NF1	SDHB	*** X
		34	157	70	18	5	4	5	1	32	14	10	2	14	51
		8.1%	37.6%	16.8%	4.3%	1.2%	1.0%	1.2%	0.2%	7.7%	3.4%	2.4%	0.5%	3.4%	12.2%
Gender															
male	164 (39.3)	16	65	25	6	2	2	3		15	5	1	1	2	21
female	253 (60.7)	18	92	45	12	3	2	2	1	17	9	9	1	12	30
Age, median, years	55.4	51.6	54.3	57.7	59	57	46.5	53.8		58.2	52.2	44.9	53	39.8	48.7
Tumor location															
esophagus	2 (0.5)		1		1										
stomach	220 (52.8)	2	76	38	14	3	3	2		31	11	8		14	20
small intestine	145 (34.8)	29	61	19	2		1	1		1	1	2	2		26
colon	8 (1.9)		3	3	1			1							
rectum	14 (3.4)	1	8	4					1						
EGIST	24 (5.8)	2	7	5		2		1			1				4
Unknown	4		1	1							1				1

*** X—KIT/PDGFRA/BRAF WT GIST cases.

**Table 3:** Novel *KIT* and *PDGFRA* mutations discovered in this study.

No.	Pat.	Age/ Sex	GIST Localization	Gene, Exon	Protein	Codons
1	111	68/M	EGIST	*KIT* ex.11	(1) p.K550N + (2) p.K558P	(1) c.1650 A>T + (2) c.1672–1673 AA>CC
2	67	71/F	Stomach	*KIT* ex.11	p.K550_W557delins QR	c.1648 A>C + c.1652–1669delins GG
3	160	59/M	Sm. Intest	*KIT* ex.11	p.M552del	c.1654–1656del
4	204	69/F	Sm. Intest	*KIT* ex.11	p.Y553D	c.1657 T>G
5	498	29/M	Colon	*KIT* ex.11	p.E554D	c.1662 A>T
6	205	57/M	Sm.Intest	*KIT* ex.11	p.V555_V559delins AT	c.1664–1676delins CCAC
7	230	31/F	Sm .Intest	*KIT* ex.11	p.V555_P577delins A	c.1664–1729delins CA
8	75	42/F	Sm. Intest	*KIT* ex.11	p.Q556_V569del	c.1665–1706del
9	168	78/F	Stomach	*KIT* ex.11	p.W557_K558delins CE	c.1671–1672 GA>TG
10	492	60/F	Colon	*KIT* ex.11	p.W557_K558delins CE	c.1671–1672 GA>TG
11	74	40/M	Stomach	*KIT* ex.11	(1) p.G565_N566 del (2) p.V569_Y570delins RR	(1) c.1692–1697del (2) c.1705–1709 delins CGGCG
12	479	9/M	Sm. Intest	*KIT* ex.11	p.Y568_L576 delins CVGS, Frameshift	c.1703–1727delins GTGTTGGGTC, Frameshift
13	77	68/F	Stomach	*KIT* ex.11	p.V569_W582del	c.1705–1746del
14	236	53/F	EGIST	*KIT* ex.11	p.P573_L576dupl	c.1719–1728dupl
15	46	66/M	Stomach	*KIT* ex.11	p.T574_R586dupl	c.1721–1757dupl
16	539	76/F	Stomach	*KIT* ex.11	p.T574_S590dupl	c.1720–1769dupl
17	464	48/M	Stomach	*KIT* ex.11	p.Q575_P577dupl	c.1722–1730dupl
18	477	52/F	Stomach	*KIT* ex.11	p.Q575_D579dupl	c.1723–1737dupl
19	61	66/F	Stomach	*KIT* ex.11	p.P577ins PF	c.1730ins ATTTCC
20	63	62/F	Sm. Intest	*KIT* ex.11	p.P577_Y578ins HLP	c.1731ins CATCTTCCT
21	161	64/M	Stomach	*KIT* ex.11	p.Y578_D579dupl	c.1732–1737dupl
22	231	68/F	Esophagus	*KIT* ex.11	p.Y578_S590dupl	c.1732–1770dupl
23	149	62/F	Stomach	*KIT* ex.11	p.D579_F584dupl	c.1735–1752dupl
24	175	50/F	Sm .Intest	*KIT* ex.11	p.K581del	c.1741–1743del
25	65	62/M	Sm .Intest	*KIT* ex.11	p.P585del, Frameshift	c.1755delC, Frameshift
26	448	62/M	Sm. Intest	*KIT* ex.17	(1) p.N819Y + (2) p.N822K (homo)	(1) c.2457A>T (2) c.2466 T>G
27	199	64/M	Stomach	*PDGFRA* ex.12	p.E563V	c.1697 A>T
28	280	60/F	Stomach	*PDGFRA* ex.18	p.A821S	c.2461 G>T
29	7	55/M	Sm. Intest	*PDGFRA* ex.18	p.C835del	c.2503–2505del
30	43	70/M	Stomach	*PDGFRA* ex.18	p.C835del	c.2503–2505del

**Table 4:** The clinicomorphological characteristics of 280 patients with GIST, for whom overall survival was monitored.

Characteristics	Number of Patients (%)
Sex	*n* = 280
Male	110 (39.3%)
Female	170 (60.7%)
Age at diagnosis	*n* = 280
Adult, (range, 21–84 years)	269 (96.1%)
median age 55,1 years	
Children (range, 9–20 years)	11 (3.9%)
median age 15,6 years	
Primary tumor location	*n* = 280
Esophagus	2 (0.7%)
Stomach	138 (49.3%)
Small intestine	107 (38.2%)
Rectum and colon	15 (5.4%)
Extra-gastrointestinal (EGIST)	17 (6.1%)
Unknown primary tumor	1 (0.4%)
Morphology	*n* = 276
Spindle cells	176 (63.8%)
Epithelioid cells	40 (14.5%)
Mixed cells	60 (21.7%)
CD117 expression	*n* = 247
Positive	240 (97.2%)
Negative	7 (2.8%)
CD34 expression	*n* = 225
Positive	170 (75.6%)
Negative	55 (24.4%)
Primary tumor size, cm	*n* = 234
0–5	47 (20.1%)
5.1–10	77 (32.9%)
>10	110 (47.0%)
Mitotic index, 50 HPF x400	*n* = 256
≤5	83 (32.4%)
5.1–10	70 (27.3%)
>10	103 (40.2%)

## Data Availability

The original contributions presented in this study are included in the article/Supplementary Materials. Further inquiries can be directed to the corresponding author.

## Supplementary Materials

The following supporting information can be downloaded at: https://www.mdpi.com/article/10.3390/cancers18203298/s1, Table S1: Forward and reverse primers for “nested” polymerase chain reaction for *KIT*, *PDGFRA* and *BRAF* genes; Table S2: Clinical characteristics of 137 GIST patients that have not follow-up data.
