# Supplementary material for: Molecular Characteristics and Survival Rates in the Patients with Gastrointestinal Stromal Tumors: A Retrospective Single-Center Study
Source: Cancers (Basel). 2026 Oct 13;18(20):3298. doi: 10.3390/cancers18203298 (PMC13702205; doi:10.3390/cancers18203298)
Supplement: Supplementary file 1 [file cancers-18-03298-s001.zip › cancers-4556716-supplementary.pdf]

Supplementary files

Table S1. Forward and reverse primers for "nested" polymerase chain reaction for *KIT*, *PDGFRA* and *BRAF* genes.

KIT	Primers	T
Exon 11	For: 5' – TAGCTGGCATGATGTGCATT – 3'	58
	Rev: 5' – TGGAAAGCCCCTGTTTCATA – 3'	58
	shFor: 5' – GGTGATCTATTTTTCCCTTTCT – 3'	60
	shRev: 5' – AAGCCCCTGTTTCATACTGAC – 3'	62
Exon 9	For: 5' – AAAAGTATGCCACATCCCAAGTG – 3'	66
	Rev: 5' – AGAAATGACATGGTCAATGTTGG – 3'	60
	shFor: 5' – ATTCAAGCACAATGGCACGG – 3'	62
	shRev: 5' – AGAAATGACATGGTCAATGTTGG – 3'	62
Exon 13	For: 5' – CTTACCTTGTTGTCTTCCTTC – 3'	60
	Rev: 5' – CCAAGCAGTTTATAATCTAGCA – 3'	60
	shFor: 5' – GAGTGCCCATTTGACAGAACG – 3'	64
	shRev: 5' – GTCATGTTTTGATAACCTGAC – 3'	58
Exon 17	For: 5' – GTTTTCTTTTCTCCTCCAACCTA – 3'	64
	Rev: 5' – CCTAGACAGGATTTACATTATGA – 3'	62
	shFor: 5' –CAGCCAGAAATATCCTCCTTAC – 3'	64
	shRev: 5' – GTACTCACGTTTCCTTTAACCA – 3'	62
PDGFRA		
Exon 12	For: 5' – TCCAGTCACTGTGCTGCTTC – 3'	62
	Rev: 5' – GCAAGGGAAAAGGGAGTCTT – 3'	60
	shFor: 5' – CATTTATAGAAACCGAGGTATG – 3'	60
	shRev: 5' –TGGAACTTACCAAGCACTAGTC – 3'	64
Exon 14	For: 5' – TGGTAGCTCAGCTGGACTGAT – 3'	64
	Rev: 5' – GGGATGGAGAGTGGAGGATT – 3'	62
Exon 18	For: 5' – TTCCTTTTCCATGCAGTGTGTCC– 3'	68
	Rev: 5' – GAAGCAACACCTGACTTTAGAGA– 3'	66
	shFor: 5' – CTCGCAACGTCCTCCTGGCACAA – 3'	74
	shRev: 5' – CTTTAGAGATTAAAGTGAAGGAG – 3'	62
BRAF		
Exon 15	For: 5' – ACCTAAACTCTTCATAATGCT – 3'	56
	Rev: 5' – ACAACTGTTCAAACTGATGG – 3'	56
	shRev: 5' – AAACTGATGGGACCCACTCC – 3'	62

Table S2. Clinical characteristics of 137 GIST patients that have not follow-up data.

Characteristics	Number of patients (%)
Sex	n= 137
Male	54 (39.3%)
Female	83 (60.6%)
Age at diagnosis	n= 137
Adult, (range, 21-84 years) median age 55,4 years	135 (98.5%)
Children (range, 9-20 years) median age 15,6 years	2 (1.5%)
Primary tumor location	n= 137
Esophagus	0 (0.0%)
Stomach	81 (59.1%)
Small intestine	39 (28.5%)
Rectum and colon	7 (5.1%)
Extra-gastrointestinal (EGIST)	7 (5.1%)
Unknown primary tumor	3 (2.2%)
Morphology	n= 114
Spindle cells	72 (63.2%)
Epithelioid cells	23 (20.2%)
Mixed cells	19 (16.6%)
CD117 expression	n= 115
Positive	106 (92.2%)
Negative	9 (7.8%)
CD34 expression	n=92
Positive	78 (84.8%)
Negative	14 (15.2%)
Primary tumor size, cm	n=55
0-5	24 (43.6%)
5.1-10	14 (25.5%)
>10	17 (30.9%)
Mitotic index, 50 HPF x400	n= 94
≤5	44 (46.8%)
5.1-10	21 (22.3%)
>10	29 (30.9 %)
